# Effect of *Portulaca oleracea* on Carcass Traits, Fatty Acid Composition, Meat Quality and Oxidative Stability of Arabi Lambs

**DOI:** 10.1002/vms3.70224

**Published:** 2025-02-07

**Authors:** Sadegh Mayahi, Morteza Chaji, Kamal Shojaeian, Ghasem Jalilvand

**Affiliations:** ^1^ Department of Animal Sciences, Faculty of Agriculture University of Zabol Zabol Iran; ^2^ Department of Animal Sciences, Faculty of Animal Science and Food Technology Agricultural Sciences and Natural Resources University of Khuzestan Ahvaz‐Mollasani Iran

**Keywords:** fatty acid composition, flavonoids, meat colour, oxidative stability, phenolic compounds

## Abstract

The aim of the present study was to investigate the effect of using *Portulaca oleracea* L. or Purslane (POL) as a source of plant antioxidants in the diet of fattening male lambs on carcass traits, meat quality and oxidative stability. Using POL in diets, meat colour characteristics were improved. The carcass traits such as weight and yield were improved (*p *< 0.05) in ration contain POL. The treatments containing POL increased the concentrations of palmitic fatty acids, alpha and gamma‐linolenic in meat (*p *< 0.05). The highest concentration of linoleic acid (C18:2 *n*‐6 *trans*) was observed in 15% POL treatment. Feeding the POL reduced the malondialdehyde concentration and increased the oxidative stability (*p *< 0.05). The results showed that POL nutrition had increased the meat quality by increasing oxidative stability, improving properties such as fatty acid composition. All of these properties, along with the increase in beneficial fatty acids in meat, have led to an increase in its nutritional value for human health.

## Graphical abstract

• Purslane has antioxidant properties and bioactive compounds.

• Protective effect on lipids and meat against oxidation, and increases meat quality, shelf life, and stabilizes the meat colour during storage, Improve the status of essential fatty acids such as the *n*‐3 family.

• Therefore, increase the quality of meat, marketability, growth performance and improve human health.

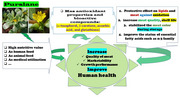

## 1 Introduction

Meat is one of the most important protein sources. The richness of meat from proteins and minerals such as iron and zinc, types of vitamins, as well as enough energy (Biesalski 2005), caused it to be categorized as the best and most complete food products. The richness of meat from valuable proteins that contain essential amino acids for the body, such as histidine, isoleucine, leucine, valine, lysine, threonine, phenylalanine, methionine and tryptophan (Górska‐Warsewicz et al. 2018), as well as lipid (Wood et al. 2008), which are considered an energy source for the body and contain fatty acids such as linoleic acid, linolenic acid and arachidonic acid (Woods and Fearon 2009), also minerals (Ahmad et al. 2018) such as phosphates and sulphates and vitamins (Pereira and Vicente 2013), especially B vitamins and carbon monoxide (glycogen), represents the value and importance of this product in human nutrition. One of the main aspects of choosing meat is the appearance and colour of the consumer. Between sensory attribute of foods, colour is perhaps the most important because if the colour is objectionable, all other sensory attributes lose significance. The consumer often uses colour as an indicator of the freshness and quality of meat. Oxidation of lipids is an automatic and inevitable process that is the leading cause of fat tissue deterioration in meat. This process directly affects the meat value and products (De Lima Júnior et al. 2013). Today, one of the massive economic problems in the meat industry is lipid oxidation. Ripoll et al. (2011) reported that the degradation of fats and proteins that cause dehydration, flavour, texture and colour of meat products results from oxidation processes in meat. Therefore, the sustainability of meat oxidation is a significant concern for all operators involved in the meat production chain, including breeders, sellers, distributors and consumers (Embuscado 2015). Many studies in recent years have been performed to improve the composition of meat fatty acids in the diet of ruminants. Changing the profiles of fatty acids by manipulating edible rations is one of the most effective methods for increasing the concentration of these acids in sheep's meat products (Deng et al. 2018). The awareness of the positive effects of some fatty acids such as vaccenic and linoleic acid conjugates, on ruminant products naturally (Ribeiro et al. 2011), as well as the beneficial effects of the secondary compounds of plants in the treatment of certain complication or diseases like cancer, oxidative damage and inhibiting microbial growth in humans (Ahmed et al. 2016; Ganesan and Xu 2017), have contributed to the enrichment of animal food with such plants. Antioxidants are substances that reduce the oxidative problems of oxidizable biomolecules such as lipids and proteins in meat products, which improve their stability and quality (Karre et al. 2013). Recent advances in the identification and use of antioxidants have enabled professionals to delay the oxidation of meat through various strategies, such as the use of natural antioxidants in animal feed and the colorimetric properties, and even improve the quality of polyunsaturated fatty acids (PUFAs) in meat and protein products (Bessa et al. 2015). Nowadays, consumers are asking for more natural foods, that is why the industry includes natural antioxidants in foods. Dietary antioxidants can be delivered to the muscle where, together with the native defence systems, they counteract the action of peroxidation (Descalzo and Sancho 2008).

*Portulaca oleracea* L. or Purslane (POL), known as a wild plant, is a good selection for study due to its high nutritive and antioxidant properties as human food, animal feed and medical utilization (Golshan‐Zoroofi et al. 2013). The bioactive compounds found in plants may have the protective effect of lipid and meat proteins against oxidation (Gladine et al. 2007). Various studies have reported the presence of various concentrations of active biological compounds such as flavonoids, alkaloids, phenol, saponin, tannin, terpenoid, *ω*‐3 fatty acids, cardiac glycoside, steroid, phlorotannin, protein, in the Purslane plant (Xiu et al. 2019; Yang et al. 2018; Sicari et al. 2018). Aside from possessing medicinal properties, this plant also supplied a source of nutritional benefits comprising being rich in *ω*‐3 fatty acids, α‐linolenic acid and antioxidants (α‐tocopherol, β‐carotene, ascorbic acid and glutathione) (Uddin et al. 2014). The antioxidant ingredients in POL include *ω*‐3 fatty acids, some phenolic compounds and total flavonoids (Simopoulos et al. 1992). There was no report on the effect of using POL in the diet of fattening lambs as a food source rich in natural antioxidants on the quality characteristics of their meat. Therefore, the purpose of this study was to investigate the effect of the different levels of POL as a plant source of antioxidants in animal feed on carcass traits, meat fatty acids composition, meat quality and oxidative stability of Arabi lambs.

## 2 Materials and Methods

All experimental procedures were carried out in Agricultural Sciences and Natural Resources University of Khuzestan (ASNRUKH) according to the care and use of agricultural animals in research and teaching guidelines (FASS 2010), and the study was approved by the ASNRUKH animal care committee (Approval no. ASD 010/1398).

### 2.1 Preparation of POL

The POL was freshly collected from agricultural fields located in Khuzestan province (Iran) and dried in the shade, crushed and then threshed.

### 2.2 Management of Animals and Diets

Twenty‐four male Arabi lambs with an average weight of 24 ± 1.5 kg and age of 150 ± 15 days were used in the present experiment. The experimental diets (Table 1) were prepared on the basis of the bodyweight using standard requirements tables (NRC 2007). The chemical composition and fatty acid composition of the diet fed to lambs are shown in Tables 1 and 2, respectively. On the basis of the chemical composition, especially the protein concentration, POL replaced alfalfa hay in the control diet. The experimental treatments included: 1—control diet (no POL), 2—diet containing 7.5% POL (replacement of 25% of alfalfa hay with POL) and 3—15% POL (replacement of 50% of alfalfa hay with POL). During the 84‐day experimental period, the lambs were individually housed in metabolic cages, fed at 8:00 and 18:00 and provided free access to drinking water.

**TABLE 1:** Feed ingredients and chemical composition of experimental diets.

Components diet (g/kg DM)	The amount of POL replaced in the diet (%)		
	Control	7.5	15
Alfalfa fodder	30	22.5	15
POL	0	7.5	15
Sugarcane top	10	10	10
Corn grain	30	30	30
Wheat bran	12.50	12.50	12.50
Barley grain	10.8	10.8	10.8
Sesame meal	5	5	5
Mineral and vitamin premix^a^	1.20	1.20	1.20
Salt	0.30	0.30	0.30
Limestone	0.20	0.20	0.20
Chemical composition (g/kg)			
Dry matter (g/kg)	880.1	884.3	878.7
Neutral detergent fibre (g/kg)	334.3	334.7	336.6
Acid detergent fibre (g/kg)	244.3	244.4	242.3
Crude protein (g/kg)	142.8	146.7	150.7
Ether extract (g/kg)	27.3	28.7	28.9
ME (Mcal/kg)	24.7	24.7	24.8

Abbreviation: POL, *Portulaca oleracea*; ME, Metabolizable energy

^a^ Each kg of premix includes: 500,000 IU vitamin A, 100,000 IU vitamin D3, 100 mg vitamin E, 400 mg antioxidants, 196,000 mg Ca, 50,000 mg Na, 18,000 mg Mg, 3000 mg Fe, 300 mg Cu, 2000 mg Mn, 3000 mg Zn, 100 mg Co, 100 mg I, 0.1 mg Se.

**TABLE 2:** Fatty acid composition (g/100 g fatty acid) of diet fed to lambs.

Item	Treatments		
	POL 0%	POL 7.5%	POL 15%
ΣFA	100	100	100
C10:0	0.45	0.39	0.41
C12:0	2.17	2.09	2.11
C14:0	5.91	5.61	5.59
C15:0	1.52	1.49	1.50
C16:0	19.75	19.97	19.68
C16:1	0.29	0.29	0.29
C17:0	1.91	1.77	1.81
C18:0	4.36	4.20	4.21
ΣPUFA	24.14	24.41	24.37
Σ*n*‐3	0.19	0.19	0.20
C18:3 *n*‐3	0.19	0.19	0.20
Σ*n*‐6	23.95	24.22	24.17
C18:2 *n*‐6	23.59	23.88	23.85
C20:4 *n*‐6	0.36	0.34	0.32
*n*‐6/*n*‐3	126.05	126.40	120.14
Σ*c*‐MUFA	39.79	39.90	40.13
C18:1 *n*‐9	39.50	39.61	39.84
ΣSFA	36.07	35.53	35.30
ΣUSFA	63.38	65.37	65.57
ΣMUSFA	39.80	41.65	41.90
USFA:SFA	1.76	1.84	1.86
PUFA:SFA	0.67	0.69	0.69

*Note*: ΣSFA = C14:0 + C16:0 + C18:0; ΣMUFA = C16:1 + C18:1 + C18:1 *trans*‐11; ΣUFA = C16:1 + C18:1 + Σ*n*‐3 + Σ*n*‐6; ΣPUFA = Σ*n*‐3 + Σ*n*‐6; Σ*n*‐3 = C18:3*n*‐3 + C20:5*n*‐3 + C22:5*n*‐3 + C22:6*n*‐3; Σ*n*‐6 = C18:2*n*‐6 + C20:4*n*‐6; *n*‐6:*n*‐3 = (C18:2*n*‐6 + C20:4*n*‐6)/(C18:3*n*‐3 + C20:5*n*‐3 + C22:5*n*‐3 + C22:6*n*‐3).

Abbreviations: ΣPUFA, sum of polyunsaturated fatty acids (Σ*n*‐6 + Σ*n*‐3); Σ*c*‐MUFA, sum of *cis*‐monounsaturated fatty acids; ΣFA, total fatty acids; MUFA, mono unsaturated fatty acids; POL, *Portulaca oleracea*; PUFA, polyunsaturated fatty acids; SFA, saturated fatty acids; USFA, unsaturated fatty acids.

### 2.3 Measuring the Chemical Composition

In order to determine the chemical composition of POL and other experimental samples after drying (90°C, 24 h) and milling with 2 mm sieve, ether extract (method 954.02—AOAC 2006), ash (electric furnace, 600°C, 4 h, method 942.05—AOAC 2006) and ADF (method 973.18—AOAC 2005) were determined by standard methods (AOAC 2005). Crude proteins were determined by the Kjeltec apparatus (FOSS Kjeltec 2300 Analyser, Sweden; method 990.03—AOAC 2006). Neutral detergent insoluble fibres (NDF) of POL were measured according to Van Soest et al. (1991). The total phenol content, the total concentration of flavonoid compounds and tannin of the ethanolic extract of dry shade POL (Table 4) were measured using the Folin–Ciocalteu method (Cicco et al. 2009). All samples were at least analysed with three replicates.

### 2.4 Carcass Traits

Animals were slaughtered at Day 84 experiment after 16–14 h fasting (Eynipour et al. 2019). Slaughtering procedure, carcass traits definition and dissection technique were followed as recommended by Fisher and Boer (1994). Live weight, mean carcass weight, carcass yield and other factors were also examined. For quality and oxidative stability analysis, meat samples were obtained between the 12th and 13th ribs.

Meat Chemical compositions of lamb include moisture (oven 103°C for 16 h; Corzo et al. 2009), crude protein (Kjeldahl method, FOSS2033, Sweden) and fat (AOAC 2005). Ash was determined according to standard methods Association of Official Agricultural Chemists (AOAC 2005). Drip loss samples of meat with a specific weight, approximately 2–2.5 cm in diameter and suspended, were stored at 2°C for 7 days. The weight difference of the samples in the first and seventh days after slaughter was used to calculate the drip loss, and the difference in weight of the samples in the first and seventh days after slaughter was used to calculate the storage loss (Hildrum et al. 2000).

### 2.5 Measurement of Meat Characteristics

The sample of *longissimus lumborum* (LL) was removed from the 12th and 13th ribs. The meat pH was measured at 1 and 24 h post‐slaughter using a pH meter (WTW: 3110, Germany) equipped with a penetrating glass electrode. To measure meat pH, pH electrode calibration was performed with standardized buffers (pH 4.0 and 7.0) and by Reis and Rosenvold (2014) method every hour. Karlsson and Rosenvold (2002) were inspired by the method for calibrating the pH meter temperature. Colour measurements were performed at 24 h postmortem on longissimus thoracis (LT) samples at the last lumbar vertebra, using a bench colorimeter chroma meter CR‐400 Konica minolta sensing (Minolta Sensing Inc, Osaka, Japan). In this experiment, the Konica minolta was calibrated with 2° standard observer to the *L*, *a*, *b* colour and illuminant D65. Colour measurements were done in terms of lightness (*L*), redness (*a*) and yellowness (*b*). Chromaticity Indices for the measurement of meat colour and the angle Hue (*H*) were calculated using the following formulas, respectively (AMSA 2012).

$$\mathrm{Chromaticity}\;\left(C\right)={\left(a^{2}+b^{2}\right)}^{1/2}$$

$$\mathrm{Hue}\left(H\right)=\mathrm{ta}n^{-1}\left(b/a\right)\times 57.29$$

Lipid oxidation was determined on meat after 1, 7 and 30 days of frozen storage using through a thiobarbituric acid reactive substance (TBARS) assay (Dabbou et al. 2014).

Measurement fatty acid: Fatty acid profile of POL (Table 2), diets (Table 3) and meat was analysed by gas chromatography device model 3800 with a 100‐m capillary column (internal diameter 25 µm). The fatty acid composition of meat and diet in this study was measured similar to the method of Berrighi et al. (2017) and Copolovici et al. (2017). Identification of fatty acid methyl esters by GLC, using standard reference number 601 from Nu‐Check Prep Inc, Elysian, MN, USA, was performed by the method proposed by Vahmani et al. (2017). The lipids were methylated using 0.5 M sodium methoxide and 5% methanolic hydrochloric acid reagents. Internal standard was L from 1 mg *c*10–17:1 methyl ester/mL toluene.

**TABLE 3:** Fatty acid composition of *Portulaca oleracea* (POL) fed to lambs.

Fatty acid	g/100 g fatty acid
C15:0	0.70
C16:0	16.15
C18:0	4.66
C16:1	0.62
C18:1 *n*‐9	6.73
C18:2	18.77
C18:3 *n*‐3	28.62
C18:3 *n*‐6	21.14
C20:0	1.13
C22:0	0.15
C24:0	1.33
ΣSFA	24.39
ΣUFA	75.88
ΣMUFA	7.35
ΣPUFA	47.39
UFA:SFA	3.11
PUFA:SFA	1.94

*Note*: ΣSFA = C14:0 + C16:0 + C18:0; ΣMUFA = C16:1 + C18:1 + C18:1 *trans*‐11; ΣUFA = C16:1 + C18:1 + Σ*n*‐3 + Σ*n*‐6; ΣPUFA = Σ*n*‐3 + Σ*n*‐6; Σ*n*‐3 = C18:3*n*‐3 + C20:5*n*‐3 + C22:5*n*‐3 + C22:6*n*‐3; Σ*n*‐6 = C18:2*n*‐6 + C20:4*n*‐6; *n*‐6:*n*‐3 = (C18:2*n*‐6 + C20:4*n*‐6)/(C18:3*n*‐3 + C20:5*n*‐3 + C22:5*n*‐3 + C22:6*n*‐3).

Abbreviations: MUFA, mono unsaturated fatty acids; PUFA, polyunsaturated fatty acids; SFA, saturated fatty acids; UFA, unsaturated fatty acids.

Atherogenicity (AI) and thrombogenicity (TI) indices were calculated by the following equations, and the method proposed by Ulbricht and Southgate (1991) also elongated activity calculated by Silva‐Santi et al. (2016) method.

$$\mathrm{AI}=\left[C12:0+\left(C14:0\times 4\right)+C16:0\right]/\left(\mathrm{Total}\;\mathrm{unsaturated}\;\mathrm{fatty}\;\mathrm{acids}\right)$$

where C12 is the percentage of lauric acid in relation to TFA; C14 is the percentage of myristic acid in relation to TFA and C16 is the percentage of palmitic acid in relation to TFA.

$$\begin{array}{ccc} & & \mathrm{TI}=\sum \left(C14:0+C16:0+C18:0\right)/(0.5\times \mathrm{cisC}18:1+0.5\\ & & \;\times \;\sum \mathrm{MSFA}+0.5\times \sum \left(\omega -6\right)+0.5\times \sum \left(\omega -3\right)+\left(\omega -3/\omega -6\right) \end{array}$$

where *ω*‐6 is fatty acids containing *ω*‐6 and *ω*‐3 is fatty acids containing *ω*‐3.

Elongase activity: ratio of 18:0/16:0

### 2.6 Statistical Analysis

Statistical analysis of the data was done in a completely randomized design with GLM procedure of SAS version 9.2, *Y_ij_* = *μ* + *T_i_* + *e_ij_*, where *Y_ij_* is observations, *μ* general mean, *T_i_* effect of treatments and *e_ij_* residual effect. The means were compared by Duncan's multiple range test at the error level of 0.05. The *Y* variables considered for analysis were carcass traits, meat quality characteristics, meat chemical composition, meat fatty acid composition and meat oxidation properties.

## 3 Results

### 3.1 Fatty Acid and Chemical Composition of POL Fed to Lambs

The main fatty acid composition of POL was palmitic (C16:0), linoleic (C18:2) and linolenic (C18:3); the most fatty acids concentration of POL is linolenic (Table 3). POL's chemical composition is detailed in Table 4. The total phenolic content (TPC), total flavonoid content (TFC) and total tannin contents of the POL plant used in the present experiment, respectively, were 313.84, 39.76 and 0.82 mg/100 g (Table 4).

**TABLE 4:** *Portulaca oleracea* (POL) chemical composition (g/kg DM) fed to lambs.

Item	POL
Dry matter	7.43
Crude protein	26.22
Organic matter	74.77
Ash	25.23
Neutral detergent fibre	50.62
Acid detergent fibre	5.43
Ether extract	7.86
Metabolic energy (MJ/kg)	2.52
Tannin (mg/100 g)	0.82
Total phenolic content (mg/100 g)	313.84
Total flavonoids content (mg/100 g)	39.76

### 3.2 Carcass Traits

The average weight of carcass traits is shown in Table 5. The highest mean hot carcass weight (22.56 kg) and the highest mean carcass efficiency (49.03%) were observed in the 15% POL treatment, significantly different from the other treatments. Visceral fat was significantly decreased in treatments containing POL (*p *< 0.05). Empty rumen weight decreased significantly with an increasing percentage of POL (*p *< 0.05). Other carcass traits were not affected by different treatments.

**TABLE 5:** Carcass traits of fattening lambs fed with different level supplementary *Portulaca oleracea* (POL).

Item	Treatments			SEM	*p* value
	POL 0%	POL 25%	POL 50%		
Live weight (g)	45.00	45.35	46.01		
Hot carcass weight (kg)	20.05^b^	21.25^a,b^	22.56^a^	0.49	0.03
Carcass efficiency (%)	44.56^b^	46.86^a,b^	49.03^a^	0.68	0.01
Head (g)	2403	2446	2469	43.16	0.57
Leg and hoof weight (g)	929.00	917.67	916.73	0.02	0.26
Skin (g)	6116	6026	6005	0.06	0.50
**Internal organs**					
Full rumen (g)	3760.0	3905.0	3892.3	181.86	0.82
Empty rumen (g)	703.57	635.63	620.00	19.63	0.05
Full reticulum (g)	158.00	160.33	163.66	4.67	0.70
Empty reticulum (g)	119.33	120.00	107.29	12.71	0.74
Full omasum (g)	234.59	252.62	245.33	9.62	0.49
Empty omasum (g)	135.00	155.00	151.67	9.54	0.34
Full abomasum (g)	274.33	287.51	288.48	16.27	0.79
Empty abomasum (g)	163.00	133.82	156.33	10.01	0.11
Full small intestine (g)	2408.00	2491.43	2442.51	24.89	0.13
Empty small intestine (g)	1063.23	1097.11	1083.48	27.15	0.68
Full large intestine (g)	824.31	824.87	825.15	9.87	0.99
Empty large intestine (g)	153.66	155.52	158.00	3.08	0.57
Lungs (g)	484.45	482.72	482.26	2.42	0.77
Spleen (g)	49.00	47.00	48.00	0.57	0.12
Liver (g)	676.32	672.00	663.98	6.03	0.36
Heart (g)	155.34	153.42	150.65	2.74	0.52
Heart fat (g)	70.00	73.00	72.32	0.96	0.14
Kidneys (g)	108.62	105.87	112.00	5.51	0.73
Kidneys fat (g)	154.13	160.00	154.54	9.77	0.90
visceral fat (g)	280.00^a^	175.67^b^	146.00^b^	25.61	0.02
**Carcass cuts**					
Tail weight (g)	1842.00	1899.12	1836.85	27.24	0.27
Neck (g)	2034.24	2042.00	2064.35	44.55	0.88

*Note*: Carcass efficiency: hot carcass weight/live weight × 100.

^a,b^ In each row, the means with different letters are significantly different (*p* < 0.05).

### 3.3 Meat Colour, Stability and Lipid Oxidation

The effect of different POL levels on the quality characteristics of fattening lamb, including pH and meat colour, is shown in Table 6. There was a significant difference between treatments (*p* < 0.05) at the pH of the first hour, but the pH of the 24 h was not significant. The lightness index (*L*) was highest in diets containing 7.5% and 15% POL. The control and treatment containing 15% POL had the highest redness index (*a*). Yellowness index (*b*) decreased significantly with the addition of POL to diets (*p* < 0.05). The lowest amount of yellowness index (*b*) was observed in the treatment containing 7.5% POL. Hue angle and chromaticity indices in POL treatments were significantly lower than in the control treatment.

**TABLE 6:** Effect of supplementation of different level of *Portulaca oleracea* (POL) on meat quality characteristics of fattening lambs.

	Treatments				
Item	POL 0%	POL 7.5%	POL 15%	SEM	*p* value^*^
pH value					
1 h	6.4^b^	6.57^a^	6.53^a^	0.04	0.04
24 h	5.67	5.59	5.64	0.03	0.22
Meat colour					
*L*	20.49^b^	23.00^a^	24.16^a^	1.51	0.025
*a*	11.02^a^	10.26^a,b^	9.84^b^	0.33	0.006
*b*	6.13^a^	4.50^b^	4.83^b^	0.35	0.0009
Chroma	12.65^a^	11.25^c^	10.56^b^	0.36	0.0031
Hue	29.01^a^	23.75^b^	21.28^b^	1.53	0.008

Abbreviations: *L*, lightness; SEM, standard error of the mean.

^a,b,c^ In each row, the means with different letters are significantly different (*p* < 0.05).

^a^ Redness.

^b^ Yellowness.

* *p *< 0.05.

### 3.4 Meat Chemical Composition of Fattening Lamb

The effect of different levels of POL on the meat chemical composition is shown in Table 7. The percentages of dry matter, protein and ether extract were not significantly different compared to the control group. However, the percentage of meat ash in different levels of POL increased compared to the control group (*p *< 0.05). The moisture content in the control treatment and the treatment containing 7.5% POL was slightly higher than the 15% treatment (*p *< 0.05).

**TABLE 7:** Effect supplementation of different level of *Portulaca oleracea* (POL) on meat chemical composition (%) of fattening lambs.

	Treatments				
Item	POL 0%	POL 7.5%	POL 15%	SEM	*p* value^*^
Moisture	72.47^a,b^	72.92^a^	70.82^b^	0.55	0.08
Drip loss	0.08	0.06	0.04	0.02	0.59
DM	28.69	28.68	29.17	1.27	0.95
CP	16.28	16.26	16.27	0.93	0.22
Ash	4.68^b^	5.05^b^	5.84^a^	0.24	0.01
EE	21.92	20.73	19.46	1.47	0.51

Abbreviations: CP, crude protein; DM, dry matter; EE, ether extract; SEM, standard error of mean.

^a,b^In each row, the means with different letters are significantly different (*p* < 0.05).

* *p < *0.05.

### 3.5 Meat Fatty Acid Composition

In the present study, dietary supplementation with POL plant had increased the concentrations of palmitic fatty acids (C16:0), gamma‐linolenic acid (C18:3 *n*‐6), alpha‐linolenic acid (C18:3 *n*‐3) and *trans* linoleic acid (C18:2 *n*‐6 *trans*) (*p *< 0.05). However, it did not have a significant effect on *cis* linoleic acid (C18:2 *n*‐6 *cis*) and other meat fatty acids. Total *n*‐3 fatty acids in POL treatment were higher than in the control treatment. Supplementation POL had no effect on the *n*‐6 to *n*‐3 ratio (Table 8).

**TABLE 8:** Effect of supplementation of different level of *Portulaca oleracea* (POL) on meat fatty acid composition in fattening lambs (g/100 g fatty acid).

Item	Treatments			SEM	*p* value
	POL 0%	POL 7.5%	POL 15%		
ΣFA	100	100	100	5.40	0.51
C10:0	0.29	0.29	0.28	0.005	0.42
C12:0	0.35	0.37	0.36	0.006	0.15
C13:0	0.16	0.19	0.17	0.007	0.20
C14:0	2.95	2.95	2.81	0.027	0.79
C15:0	0.48	0.49	0.47	0.010	0.64
C16:0	20.67	23.25	22.83	0.29	0.0002
C17:0	1.17	1.18	1.14	0.032	0.80
C18:0	14.67	14.75	14.37	0.217	0.29
C20:0	0.25	0.27	0.27	0.013	0.18
ΣMUFA					
C16:1	2.27	2.33	2.25	0.034	0.16
C17:1	1.23	1.19	1.17	0.030	0.80
Σ*cis*‐MUFA					
C18:1 *n*‐9 *cis*	28.16	24.93	27.22	2.20	0.43
C18:2 *n*‐6 *cis*	10.17	10.20	9.59	0.327	0.91
Σ*trans*‐MUFA					
C18:1 *n*‐9 *trans*	1.50	1.52	1.43	0.027	0.59
C18:2 *n*‐6 *trans*	0.50^c^	0.53^b^	0.54^a^	0.005	0.0002
C18:3 *n*‐3	0.37^b^	0.42^a^	0.41^a^	0.037	0.001
C18:3 *n*‐6	2.57^b^	2.77^a^	2.73^a^	0.007	0.001
C20:0	0.25	0.27	0.27	0.013	0.18
C20:1	1.34	1.35	1.26	0.018	0.53
C21:0	0.15	0.16	0.15	0.013	0.91
C20:2	1.29	1.31	1.24	0.033	0.83
C22:0	2.01	2.10	1.99	0.085	0.61
C20:3 *n*‐6	0.40	0.39	0.36	0.023	0.72
C20:3 *n*‐3	1.09	1.13	1.07	0.039	0.54
C20:4 *n*‐6	2.26	2.23	2.19	0.033	0.41
C20:5 *n*‐3	1.26	1.27	1.19	0.011	0.55
C22:6 *n*‐3	0.46	0.46	0.46	0.025	0.77
C24:0	0.37	0.37	0.37	0.018	0.59
C24:1	1.39	1.40	1.35	0.026	0.54
ΣSFA	43.78^b^	46.62^a^	45.49^a^	0.38	0.0004
ΣUSFA	56.25	53.42	54.46	2.46	0.55
ΣMUSFA	35.89	32.72	34.68	2.21	0.45
ΣPUFA	20.36	20.71	19.77	0.33	0.44
Σ*n*‐3	3.17	3.28	3.13	0.05	0.003
Σ*n*‐6	15.89	16.12	15.40	0.29	0.88
*n*‐6/*n*‐3	5.01	4.92	4.93	0.03	0.08
USFA:SFA	1.28	1.15	1.20	0.04	0.15
PUSFA:SFA	0.47	0.44	0.43	0.006	0.04
Atherogenicity index	0.59	0.68	0.64	0.03	0.20
Thrombogenicity index	0.91	1.06	0.98	0.06	0.27
Elongase activity	56.12	60.55	62.40	1.29	0.11

*Note*: ΣSFA = C14:0 + C16:0 + C18:0; ΣMUFA = C16:1 + C18:1 + C18:1 *trans*‐11; ΣUFA = C16:1 + C18:1 + Σ*n*‐3 + Σ*n*‐6; ΣPUFA = Σ*n*‐3 + Σ*n*‐6; Σ*n*‐3 = C18:3*n*‐3 + C20:5*n*‐3 + C22:5*n*‐3 + C22:6*n*‐3; Σ*n*‐6 = C18:2*n*‐6 + C20:4*n*‐6; *n*‐6:*n*‐3 = (C18:2*n*‐6 + C20:4*n*‐6)/(C18:3*n*‐3 + C20:5*n*‐3 + C22:5*n*‐3 + C22:6*n*‐3). Atherogenicity index: [(C12:0 + (4 × C14:0) + C16:0)]/(ΣMUFA + Σ*ω*‐3 + Σ*ω*‐6). Elongase: ([(C18:0 + C18:1 *cis ω*‐9)/(C16:0 + C16:1 + C18:0 + C18:1 *cis ω*‐9] × 100). Thrombogenicity index: Σ(C14:0 + C16:0 + C18:0)/(0.5 × *cis* C18:1 + 0.5 × ΣMSFA + 0.5 × Σ(*ω*‐6) + 0.5 × Σ(*ω*‐3) + (*ω*‐3/*ω*‐6).

Abbreviations: ΣFA, total fatty acids; ΣPUFA, sum of polyunsaturated fatty acids (Σ*n*‐6 + Σ*n*‐3); Σ*c*‐MUFA, sum of *cis*‐monounsaturated fatty acids; ΣMUFA, sum of monounsaturated fatty acids = (Σ*t*‐18:1 + Σ*c*‐MUFA); Σ*t*‐MUFA, sum of *trans*‐monounsaturated fatty acids; *c*, *cis*; MUFA, mono unsaturated fatty acids; PUFA, polyunsaturated fatty acids; SFA, saturated fatty acids; *t*, *trans*; UFA, unsaturated fatty acids.

^a,b,c^In each row, the means with different letters are significantly different (*p* < 0.05).

### 3.6 Meat Oxidation Changes

The effect of storage period on meat malondialdehyde concentration in lambs fed with different levels of POL is shown in Table 9. Feeding the lambs with POL significantly reduced the malondialdehyde concentration of the meat of these animals up to 30 days of storage. In other words, by adding POL to the diet of lambs, the inhibition of oxidation increased and consequently, the concentration of malondialdehyde decreased (Table 9).

**TABLE 9:** The effect of storage period on concentration of malondialdehyde in lamb fed with different level supplementary *Portulaca oleracea* (POL) (mg/kg).

	Treatments				
Storage time (day)	POL 0%	POL 7.5%	POL 15%	SEM	*p* value^*^
1	0.683^a^	0.671^b^	0.666^b^	0.003	0.007
7	1.865^a^	1.853^a,b^	1.840^b^	0.005	0.008
30	6.47^a^	6.35^b^	6.30^b^	0.026	0.0008

Abbreviation: SEM, standard error of mean.

^a,b^In each row, the means with different letters are significantly different (*p* < 0.05).

* *p < *0.05.

## 4 Discussion

### 4.1 Phenolic Content

The TPC in the dried leaves of *P. oleracea* was reported to be 260.19 mg/100 g (Uddin et al. 2014) and 300 mg/100 mL (Lim and Koah 2007). El Kashef et al. (2018) reported that the total amount of phenol and flavonoids leaves *P. oleracea* in ethanol extract, respectively, was 239.63 ± 34.46 and 34.66 ± 4 mg/100 g. Moreover, in other studies, the TPC of the leaf is about 600 mg/100 g (Cai et al. 2004) which is close to the present experimental data.

### 4.2 Carcass Traits

Increasing the carcass weight of lambs fed the POL plant is of high importance in this study because it means improving meat performance in these animals. It has been shown that when visceral fat is reduced, carcass yield will increase (Salinas‐Rios et al. 2014). Meat performance has an inverse relationship with the amount of visceral fat. This result is confirmed by Partida and Martinez (2010). They reported that the percentage of muscles decreased with increasing body fat. It seems that the increase in carcass weight in the POL‐containing treatments in this study is a result of the reduction in visceral fat content by the POL antioxidant compounds. Wang et al. (2014) showed that dietary polyphenols decrease lipogenesis and increase lipolysis, which reduces body fat. Studies of secondary plant compounds have shown that antioxidants in the plant, such as kaempferol, decrease the expression of lipid transcription factors and decrease triglyceride synthesis genes; they also increase the genes involved in lipolysis, and this event causes increased fat lipolysis (Park et al. 2012). The existence of quercetin and kaempferol as free flavonoids in the Purslane plant has been reported by different researchers (Erkan 2012). Contrary to the results of the present study, Cimmino et al. (2018) did not observe a significant difference in carcass weight and yield after the slaughter of Saanen goats fed diets containing polyphenol supplements. Hukerdi et al. (2019), by adding olive leaf as a rich source of phenolic compounds to the diet of fattening goats, did not observe a significant difference in any of the carcass traits. Although the addition of POL to the diet did not affect other characteristics of carcasses of Arabi lambs, given the high nutritional value of POL and the low price of Preparation this plant, it can have economic advantages in finished feed price and livestock products.

### 4.3 Meat Colour Stability and Lipid Oxidation

Optimal pH values in sheep meat have been reported, in the range of 6.41–6.55 for the time, or a time of slaughter (zero time) (Chikwanha et al. 2019) and 5.5–5.8 for 24 h after slaughter (Jiang et al. 2015; Majdoub‐Mathlouthi et al. 2013). In this range, the probability of forming dark, firm and dry meat in sheep is low. In the present study, the mean pH decreased from 6.5 in the first hour to 5.63 in 24 h. In general, average or typical values of decreased pH in the carcass after 24 h compared to the slaughter time indicate that quality indicators such as water holding capacity, taste, colour and texture of meat are satisfactory (Bezerra et al. 2016). It is proven that the effect of nutrition on meat pH is related to muscle glycogen levels and the mechanism of glycogen conversion to lactic acid (Immonen et al. 2000). Increasing the metabolic activity in the organism increases the production of free radicals (Halıcı et al. 2012). Free radicals increase the activity of adenosine monophosphate protein kinase (AMPK) in the tissues and thus hurt, harm, on glycogen synthesis and conversion to lactic acid in muscle tissue (Scheffler et al. 2011). Diet antioxidants affected the meat pH by eliminating free radicals and reducing the activity of the AMPK enzyme. Dietary antioxidants, by reducing the activity of the AMPK enzyme, on the one hand, increase glycogen synthesis and, on the other hand, reduce its conversion to lactic acid, thereby delaying the pH of meat (Imik et al. 2018). Higher meat pH after slaughter in the diet containing POL in this study may indicate this fact that POLs with vigorous or intense antioxidant activity (Table 4) may affect the pH of meat at slaughter and maintain the pH in the levels that are high, which preserves the quality characteristics of the meat. In the present study, no significant difference was observed between different treatments in pH at 24 h (Table 6). The pH of time 24 h significantly affects the organic properties (colour, taste and flavour) and processing technology (water holding capacity and shelf life) (Yarali et al. 2014). The results of the present experiment at pH at 24 h were in agreement with the results of Dabbou et al. (2018) in growing rabbits. They showed that dietary contain flavonoids (POL contains flavonoids, Table 4) did not affect the pH value of the meat of growing rabbits. According to the results of the present experiment, Simitzis et al. (2019) did not observe significant changes in meat quality characteristics such as pH at time 24 when supplementing flavonoids in sheep diets (Simitzis et al. 2019). Contrary to the findings of the present experiment, in the study of Muela et al. (2014), the addition of flavonoid‐rich citrus extracts (mainly orange, quercetin and rutin, 150 µg/kg) to the lamb diets did not affect pH at zero time. Differences in meat quality parameters can be attributed to the type and amount of flavonoids complement periods and animal species (Simitzis et al. 2019).

The long‐term supplementation with POL had a significant effect on the colour indexes (*L*, *a* and *b*) of LD muscle (Table 6). Myoglobin is a haem protein and is responsible for the colour of meat. Oxidation of the central iron atom in the haem group is responsible for changing the colour of the flesh from light red to dark brown. Because with the oxidation of the iron atom, red oxymyoglobin is converted to brown metmyoglobin; thus, the colour of the meat changes from light red to dark brown (Faustman et al. 2010). The highest correlation coefficient between meat quality and the colour is generally expressed for changes in the redness index (*a*). Discoloration of fresh meat is associated with the loss of nutrients and other critical sensory properties, which can have significant detrimental effects on the digestion and availability of nutrients, which are considered today in meat science (Bekhit et al. 2019). One of the reasons affecting the brightness (*L*) and redness in the present study is probably due to flavonoid and polyphenolic antioxidant compounds in POL (Table 4). It seems that the natural antioxidants existent in plants such as POL, using enzyme‐based methemoglobin reducing systems, preserve the natural colour of the meat (Liu et al. 2015). It seems that POL, with its secondary polyphenolic compounds such as flavonoids and tannins (Table 4), has maintained the meat brightness index in the present study. Luciano et al. (2009) with investigation of the effect of diets containing tannins on meat colour, suggesting that lower levels of pigments (haemoglobin and myoglobin) could improve meat colour brightness in sheep‐fed diets containing Tannin. Controlling oxidation and thus reducing the expression of myoglobin dye causes preservation of the meat brightness (Nieto and Ros 2012). Many researchers have attributed the reduction in lipid oxidation and reduced meat discoloration to phenolic compounds in plant products (Natalello et al. 2020; Emami et al. 2015). For example, herbs such as thyme and rosemary (containing natural antioxidants) in the diet reduced the lipid oxidation and the stability of the meat colour (Nieto and Ros 2012; Serrano et al. 2014). Moreover, Fan et al. (2019) showed that the amount of *L* (brightness) was significantly higher for the two higher *P. oleracea* compared to other treatments. Fruet et al. (2019) reported that brightness index values in beef‐fed diets containing rosemary (containing potent antioxidants similar to POL) were significantly higher than the control treatment. Aligned with the results of the present experiment, adding grape seed and rosemary extract as natural antioxidants to beef and pork (Rojas and Brewer 2008) or pomegranate seed pulp to goat meat (Emami et al. 2015) causes a decrease in yellowness index (*b*). Fan et al. (2019) showed that the additions of *P. oleracea* extract during freezing and storage of pork maintained the meat redness index (*a*) compared to the group without *P. oleracea*. This may indicate the effect of antioxidants in POL in preventing the oxidation of meat lipids. The low values of colour angle (Hue) in meat indicate the resistance of meat compounds to the formation of metmyoglobin and reduce oxidation in muscle (Luciano et al. 2011). It is believed that the larger colour angle (Hue) indicates the brown and undesirable colour of the meat (Ghris 2007), and low levels of chroma include lower meat colour intensity (King et al. 2012). Consistent with the findings of the present study, Luciano et al. (2009) showed that supplementation of tannin (regarding existence of tannin in POL) into the diet reduced the meat colour angle index of lambs. It seems that reducing the formation of methemoglobin, as well as decreasing the concentration of haem in the meat of lambs fed diets containing phenolic compounds (such as tannins), may be the reason for the decrease in the amount of colour angle (Luciano et al. 2009) in lambs fed POL as the plant contains phenolic substances in this study.

### 4.4 Meat Chemical Composition

Agreement our results, Cimmino et al. (2018) and Yakan et al. (2016) did not see any significant differences in the chemical composition of goat meat when supplementing phenolic compounds and vitamin C as plant antioxidants. Ezekwe et al. (2011) reported that adding dried portulaca to the pork diet increased the muscle ash content compared to the control group. Phenolic compounds such as tannins and flavonoids (found in POL, Table 4) have osteogenic activity (ossification) and improve the absorption and deposition of minerals (calcium, phosphorus, magnesium, manganese, iron and copper) in bone (Sahin et al. 2006; Mirdehghan and Rahemi 2007). The meat moisture of Arabi lamb in the present study is similar to the amounts reported by other researchers for some of the lambs’ muscles (Ekiz et al. 2009; Liu et al. 2018). According to the findings of this study, it has been reported that tea catechins reduce the moisture content of *longissimus dorsi* muscle in goats (Tan et al. 2011). Researchers believe that improving nitrogen receive and storage by altering ruminal fermentation by phenolic compounds such as flavonoids can be one of the most effective mechanisms in reducing meat moisture (Tan et al. 2011). However, the effect of flavonoids in reducing goats meat moisture (Cho et al. 2010) and lambs (Andres et al. 2014) fed quercetin supplementation, as well as orange supplementation in lambs (Simitzis et al. 2019), was not observed. Most studies on the effects of plant flavonoids in the diet of fattening lambs did not show any effect on meat moisture (Liu et al. 2018). It is believed that the reason for these differences in meat moisture results may be the different types of muscles in measuring meat moisture (North et al. 2019).

### 4.5 Meat Fatty Acid Composition

The effect of plant polyphenols on the manipulation of ruminal biohydrogenation, and thus the composition of meat's fatty acid, has been reported in previous studies (Yusuf 2014; Odhaib et al. 2018). According to the results of this study, Karami et al. (2013) found that increased concentrations of alpha‐linolenic acid (C18:3 *n*‐3) in goat meat were associated with higher levels of α‐linolenic acid in fed canola oil. De Oliveira Lima et al. (2018) also showed that feeding sunflower cake as a rich source of linoleic acid (C18:2 *n*‐6) increased the level of this fatty acid in Santa lambs. The increase in the number of fatty acids such as palmitic in meat is related to the decrease in the activity of the stearoyl CoA desaturase; it is an endoplasmic reticulum stearoyl CoA desaturase that catalyses the biosynthesis of unsaturated fatty acids with a double bond of saturated fatty acids that are synthesized in the body or taken from the diet (Forrester‐Anderson et al. 2006). Stearoyl CoA desaturase is a lipogenic key enzyme that converts saturated fatty acids such as palmitic acid (C16:0) to unsaturated fatty acids such as oleic acid. Polyphenols in grape shell extract are able to regulate lipogenesis‐mediated genes, such as the Stearoyl CoA desaturase (Tutino et al. 2020). Dietary unsaturated fatty acids, such as linoleic acid (C18:2 *n*‐6), reduce the enzyme stearoyl CoA desaturase (Ntambi 1999). Due to the high level of unsaturated fatty acids in *P. oleracea*, it seems that diets containing this plant are a suitable substrate for the enzyme Stearoyl CoA desaturase for the synthesis of palmitic fatty acid. On the other hand, the concentration of gamma‐linolenic acid (C18:3 *n*‐6) and alpha‐linolenic acid (C18:3 *n*‐3) in diets containing POL increased significantly compared to the control. The results of various studies indicate that higher concentrations of PUFA with *ω*‐3 (PUFA *n*‐3) double bonds in animal feed lead to more excellent or a greater storage of alpha linolenic acid (C18:3 *n*‐3) in meat, although most of this fatty acid in the rumen is hydrogenated to stearic acid, a percentage escapes ruminal fermentation and is absorbed in the small intestine (Berrighi et al. 2017). Therefore, perhaps the reason for the increase in these meat fatty acids in the lamb is the higher concentration of these unsaturated fatty acids in POL (Table 4). The ratio of unsaturated fatty acids to saturated fatty acids (USFA/SFA) is widely used to assess the nutritional meat value (Wood et al. 2004). The composition of fatty acids in lamb, due to the higher unsaturated fatty acids in this study, can be more important in human health and product quality. Contrary to the results of the present study, Cimmino et al. (2018) showed that the addition of polyphenols extracts of the or an olive mill to lambs diet resulted in a significant increase in MUFA concentration with decreasing SFA compared to the control group, whereas agreeing with the results of the present study was not observed effect on PUFA.

Delaying the oxidation of *ω*‐3 unsaturated fatty acids can be induced by natural flavanols, such as quercetin glycosides (Huber et al. 2009). According to the results of the present experiment, Lup et al. (2018) showed that the addition of camelina oil (as a rich source of alpha‐linolenic amino acid) to the lambs diets reduced the *ω*‐6 (*n*‐6) to *ω*‐3 (*n*‐3) ratio. One of the essential, critical and crucial aspects of the nutritional meat value is the ratio of *ω*‐6 to *ω*‐3, which should not be greater than 4 (Lup et al. 2018). In the present study, the percentage of *n*‐3 increased due to the higher level of linolenic acid, but the amount of *n*‐6 was not affected by different percentages of POL. Therefore, the ratio of *n*‐6 to *n*‐3 in samples containing POL was not affected (Table 8). It is increasing, or it was increasing the content of meat PUFA (*n*‐3 PUFA), which improves (decreases) *ω*‐6 to *ω*‐3 ratio in the human diet and is considered to be an essential point in the process of increasing the meat value (Zdunczyk and Jankowski 2013). This can be achieved by supplementing diets rich in *ω*‐3 amino acids such as POL. The composition of meat fatty acids is one of the most critical aspects of increasing the quality and nutritional value, especially for its consumers (Liu et al. 2018). One of the meat's desirable traits is its high unsaturated fatty acids content (Yarali et al. 2014). There is ample evidence that shows long‐chain *ω*‐3 unsaturated fatty acids, which play an essential role in preventing and reducing the risk of chronic disease in humans (Nguyen et al. 2018). Cao et al. (2012) showed that replacing dietary saturated fatty acids with PUFAs reduces the risk of heart disease by lowering the density of low‐density lipoprotein cholesterol. Typically, or usually, ruminant meat contains small amounts of unsaturated fatty acids and large amounts of saturated fatty acids that these imbalances in the diet make people susceptible to cardiovascular disease. Two essential fatty acids linoleic and linolenic, which are supplied only by diet, play a crucial role in human health and are precursors for synthesizing long‐chain fatty acid homologs *n*‐6 and *n*‐3 (Girard et al. 2016). One of the highlights of the present study was the high level of linolenic fatty acid in both *cis* and *trans* forms, as well as linoleic acid in treatments containing POL, which indicates the positive effect of POL unsaturated fatty acids on the composition of meat fatty acids.

### 4.6 Meat Oxidation

This indicates an increase in meat shelf life (Cunha et al. 2018). According to the present experiment, a significant decrease in the concentration of malondialdehyde in meat pork has been reported during storage, with the addition of POL (Fan et al. 2019) or *Ginkgo biloba* leaf powder (Zhang et al. 2016) which contain antioxidant compounds such as phenols and flavonoids. Improvement of antioxidant meat status in lambs after feeding plants rich in phenolic compounds and other antioxidants has been reported in several studies (Vasta and Luciano 2011; Hukerdi et al. 2019). Karami et al. (2010) reported that when sheep were fed diets rich in vitamin E and plants containing substances containing high phenolic compounds (such as POL), the concentration of malondialdehyde decreased in all treatments, which this factor protected the meat from lipid oxidation. Studies show that phenolic compounds can have a reducing effect on meat lipid oxidation (Cimmino et al. 2018). So, reducing meat lipids oxidation and further reduction of malondialdehyde in the present study may be due to phenolic and flavonoid POL compounds (Table 4). These can be beneficial effects of using POL in the diet of fattened lambs for the health of human products. Because various studies have shown that diet is the leading cause of changes in the composition of lamb meat fatty acids (Wood et al. 2004; Schmid et al. 2006).

## 5 Conclusions

This study showed that the use of POL in the diets of fattening lambs was able to increase meat quality, shelf life and reduce meat oxidation. POL supplement stabilized the meat colour during storage. The use of this plant, which is rich in antioxidants such as flavonoids, to maintain the health of lambs and improve the status of essential fatty acids such as the *ω*‐3 family, as well as the oxidative stability of meat that is consumed by humans, it will be evaluated positively. In general, the richness of POL in fatty acids such as *ω*‐3 and *ω*‐6 and linoleic acid caused that feeding this plant to fattening lambs to increase the quality of meat in terms of colour and other properties affecting human health and marketability.

## Author Contributions

**Sadegh Mayahi**: conceptualization, formal analysis, investigation, writing–original draft. **Morteza Chaji**: conceptualization, data curation, formal analysis, funding acquisition, investigation, methodology, project administration, supervision, validation, writing–review and editing. **Kamal Shojaeian**: conceptualization, data curation, formal analysis, visualization. **Ghasem Jalilvand**: conceptualization, data curation.

## Ethics Statement

The authors confirm that the ethical policies of the journal, as noted on the journal's author guidelines page, have been adhered to and the appropriate ethical review committee approval has been received. The authors confirm that they have followed EU standards for the protection of animals used for scientific purposes [and feed legislation, if appropriate].

## Conflicts of Interest

The authors declare no conflicts of interest.

### Peer Review

The peer review history for this article is available at https://publons.com/publon/10.1002/vms3.70224.

## Data Availability

The data that support the findings of this study are available from the corresponding author upon reasonable request.
